# Patient perceptions of psoriatic disease in Japan: Results from the Japanese subgroup of the Understanding Psoriatic Disease Leveraging Insights for Treatment (UPLIFT) survey

**DOI:** 10.1111/1346-8138.16423

**Published:** 2022-05-27

**Authors:** Hideshi Torii, Mitsumasa Kishimoto, Masayuki Tanaka, Hidehisa Noguchi, Siddharth Chaudhari

**Affiliations:** ^1^ Division of Dermatology Tokyo Yamate Medical Center Tokyo Japan; ^2^ Department of Nephrology and Rheumatology Kyorin University School of Medicine Tokyo Japan; ^3^ Amgen K.K. Tokyo Japan

**Keywords:** Japan, population survey, psoriasis, quality of life, treatment goal

## Abstract

The population‐based Understanding Psoriatic Disease Leveraging Insights for Treatment (UPLIFT) survey was designed to better understand patient and dermatologist perceptions of the disease burden of psoriasis (PsO) and their treatment expectations. UPLIFT was a cross‐sectional, quantitative, online survey conducted in Europe, North America, and Japan between 2 March and 3 June 2020. In Japan, 391 patients reporting a diagnosis of PsO and/or psoriatic arthritis (PsA) were surveyed (75% had PsO alone, 23% had PsO and PsA, and 2% had PsA alone). Self‐reported body surface area (BSA) data were available for 309 Japanese patients, with the majority (80%) reporting PsO‐involved BSA ≤3 palms. Current symptoms of PsO were rated as moderate or severe by 43% of Japanese patients with BSA ≤3 palms, and severe by 44% of patients with BSA 4–10 palms. PsO frequently occurred in ≥1 special areas, most commonly the scalp in 76% of Japanese patients with BSA ≤3 palms, and ≥90% of those with BSA ≥4 palms. Furthermore, musculoskeletal symptoms in 42% of patients with PsO alone were suggestive of PsA. Whereas Japanese patients with BSA ≤3 palms mainly reported receiving topical therapy alone (34%) or no treatment (32%), 64% patients with BSA 4–10 palms reported receiving systemic therapy. Overall, 21% of Japanese patients with self‐perceived mild symptoms of PsO and 48% of patients with special area involvement experienced at least a moderate impact of disease on quality of life (Dermatology Life Quality Index score >5). Moreover, patients and dermatologists differed in their perceptions of determinants of PsO severity and treatment, and office visit discussions. In general, these findings from the Japanese subgroup of the UPLIFT survey demonstrated that a high proportion of patients perceived their symptoms to be moderate or severe irrespective of the level of skin involvement, suggesting a persistent unmet treatment need.

## 1 INTRODUCTION

Psoriasis (PsO) is a chronic, systemic inflammatory disease that is characterized by erythematous skin plaques and associated with a number of comorbidities, including psoriatic arthritis (PsA).^1^, ^2^, ^3^ Without appropriate treatment, PsO can have a major impact on patients' quality of life (QoL).^1^, ^2^, ^4^, ^5^

In general, topical therapies are recommended for patients with mild or localized disease, and systemic therapies (oral medication and/or biologics) are considered for patients with more severe disease, and are routinely prescribed for patients who also have PsA.^1^, ^6^, ^7^, ^8^, ^9^ Systemic therapies can also be used for localized PsO involving special areas, such as the scalp, palms, soles, and genitals,^1^, ^10^ and in patients with mild‐to‐moderate PsO who have musculoskeletal symptoms (although joint involvement often goes unrecognized).^11^, ^12^ When defining disease severity and selecting treatment, current guidelines and international expert consensus statements recommend considering symptoms, objective measures of disease severity, response to topical treatment, involvement of areas associated with high disease burden that negatively affects patient QoL (e.g., joint involvement, and involvement of the “special areas” of the face, palms, soles, scalp, nails, and genitalia), and impact of psoriatic disease on patient QoL.^8^, ^9^, ^13^, ^14^

The Multinational Assessment of Psoriasis and Psoriatic Arthritis (MAPP) survey, a population‐based survey conducted in Europe and North America in 2012, revealed that even patients with limited skin involvement experienced substantial disease burden and impact on QoL, and many patients with PsO and/or PsA were undertreated (i.e., receiving no treatment or topical treatment alone).^5^ Since the MAPP survey was conducted, new systemic therapies with different mechanisms of action and better efficacy have been approved, and these have changed the treatment landscape of psoriatic disease in Japan.^9^, ^15^, ^16^, ^17^ The recent Understanding Psoriatic Disease Leveraging Insights for Treatment (UPLIFT) survey conducted in 2020 reported that globally there was still persistently high disease burden and undertreatment of patients with PsO and/or PsA.^11^

In Asian countries, including Japan, patients with PsO may experience high levels of social stigma and discrimination for cultural and socioeconomic reasons,^18^ and the impact of PsO on patient QoL in these countries may be even greater than that observed in Western countries. The limitations of topical therapies, including inconvenience and difficulty with application (particularly for patients with PsO in special areas), as well as perceived poor efficacy and fear of corticosteroid‐associated adverse effects,^2^, ^19^, ^20^ may contribute to low satisfaction with topical treatment observed in surveys of Japanese patients,^9^, ^21^ and poor QoL. Low treatment satisfaction has also been reported by Japanese patients with moderate‐to‐severe PsO in a survey conducted between 2015 and 2016, in which the proportions of patients receiving topical drugs, oral drugs, and biologics were 82.6%, 52.9%, and 25.4%, respectively.^22^, ^23^

To provide further insight into the status of PsO management in Japan, we present key patient‐reported data from the Japanese subgroup of patients from the UPLIFT survey, with particular focus on PsO burden and current treatments in patients with mild‐to‐moderate disease. We also describe the factors that are perceived to contribute to disease severity by both patients and dermatologists, and their treatment expectations.

## 2 METHODS

### 2.1 Survey design

The methodology and design of the UPLIFT survey have been reported previously.^11^ The survey was developed with input from a steering committee consisting of international experts in dermatology and rheumatology, and conducted by the AplusA Bell Falla (AABF) between 2 March and 3 June 2020. The protocol was approved by a central institutional review board (IRB) in the USA to comply with human subject research requirements. The IRB provided oversight over the conduct of the study in all participating countries to ensure compliance with all national standards. Therefore, the study was conducted in accordance with applicable Japanese laws and regulations, Good Clinical Practice, and the principles of the Declaration of Helsinki. Informed consent was obtained before participation in any survey procedures.

UPLIFT was a cross‐sectional, quantitative, web‐based survey conducted in Canada, France, Germany, Italy, Japan, Spain, the UK, and the USA, and was administered in the respective local language(s). The time required to complete the survey was approximately 25 min for the patient survey and 30 min for the physician survey. The surveys were developed in English and translated into Japanese for participants residing in Japan.

To recruit patients for the survey, AABF partnered with Dynata, Sermo, and Survey Healthcare, which are external fieldwork organizations specializing in internet‐based panels across a range of diseases, including PsO and PsA. All panels are moderated with native language panel support and country‐specific reward choices. The primary fieldwork partner in Japan was Dynata, who also subcontracted recruiting to WiseWorks, Cint, Kantar Lightspeed, AIP, Paneland, dataSpring, and Quest to achieve the total sample required for the survey.

For Japanese participants, the target sample size was 400 patients and 50 dermatologists. Random sampling methods were employed to identify a representative patient sample from a general population online panel of adults, who actively opted to participate in the research panel in each country, and to source dermatologists from representative panels of physicians. Additional samples were sourced from web intercept (pop‐up or other advertising). Samples from the various panels were compared using digital fingerprint technology to prevent duplication of panelists. Patient recruitment was stratified to general population demographics based on sex, age, and region for the respective countries. Dermatologists were qualified based on a screening questionnaire. Honorarium for patients and dermatologists were 219 JPY and 5599 JPY, respectively.

### 2.2 Eligibility criteria

Adults (aged ≥18 years) with a self‐reported health‐care provider diagnosis of PsO and/or PsA were eligible to participate in the patient survey. The physician survey included dermatologists who reported spending ≥50% of their professional time directly treating patients and ≥50% of office visits specifically on medical dermatology, and seeing ≥20 patients with psoriasis in a typical month, including adults with plaque PsO.

### 2.3 Assessments

In alignment with the overall population, custom and validated patient assessments in this subanalysis included respondent demographics and clinical characteristics; presence of PsO in all areas and in areas of high disease burden (i.e., genital, nail, scalp, palms, and soles); musculoskeletal/PsA symptoms; Psoriasis Epidemiology Screening Tool (PEST) scores;^24^ self‐reported skin involvement by PsO‐involved body surface area (BSA) (assessed by number of palms, based on the palm width of the hand including fingers); self‐rated current disease severity on a visual analog scale (1–3 = mild, 4–6 = moderate, 7–10 = severe); Dermatology Life Quality Index (DLQI); and current treatment.^11^ Treatment options asked in the survey were topical therapies (over‐the‐counter or prescription topical medications), oral therapies (over‐the‐counter or prescription oral medications), biologic therapy (injectable or i.v. medications), phototherapy or light‐based treatment, and other (the respondent was asked to specify).

Patient and dermatologist alignment on determinants of disease severity, treatment priorities, and treatment goals was also assessed; respondents were asked to rank their top three contributing factors for each metric. Patients and dermatologists were also asked about their perceptions of office visit discussions.

### 2.4 Analyses

Patient and dermatologist survey response results were summarized descriptively.^11^ Data for patient‐rated current disease severity, current treatment, and DLQI were analyzed by level of BSA skin involvement (i.e., ≤3 palms, 4–10 palms, or >10 palms). An analysis of patients with limited skin involvement (BSA ≤3 palms) and PsO in at least one special area (i.e., scalp, nails, palms and/or soles, face, or genitals) was used to evaluate patient‐rated current disease severity, current treatment, and DLQI score. Data were summarized using descriptive statistics without data imputation using SAS Enterprise Guide 7.15 HF9 software (SAS).

## 3 RESULTS

### 3.1 Prevalence of PsO and PsA

In Japan, of the 58 497 participants who responded (i.e., participants who clicked on the survey link), 52 326 answered the diagnostic screening question for PsO and PsA; 391 respondents self‐reported a health‐care provider diagnosis of PsO and/or PsA and met inclusion criteria, and were included in the final sample. The estimated population prevalence of PsO and/or PsA in the screened patients was 2%.^11^ As shown in Table 1, most Japanese patients reported a diagnosis of PsO alone (75%), and 25% reported PsA with or without PsO (23% and 2%, respectively).

**TABLE 1:** UPLIFT demographics and patient characteristics for the Japanese subgroup

Characteristic	*n* = 391
Mean age, years	46.6
Sex, *n* (%)	
Male	227 (58.1)
Female	164 (41.9)
Diagnosis, *n* (%)	
PsO alone	293 (74.9)
PsO + PsA	91 (23.3)
PsA alone	7 (1.8)
Comorbidities, *n* (%)	
Arthritis^a^	98 (25.1)
Cancer	83 (21.2)
Depression	111 (28.4)
Diabetes	91 (23.3)
Heart disease	72 (18.4)
Hypertension	139 (35.6)
Inflammatory bowel disease^b^	49 (12.5)
Liver disease	80 (20.5)
PsO in special areas^c^, *n* (%)	
Scalp	184 (47.9)
Face	100 (26.0)
Palms	78 (20.3)
Soles	66 (17.2)
Nails	60 (15.6)
Genitals	38 (9.9)
PsO symptoms^d^, *n* (%)	
Itching	348 (90.6)
Redness	330 (85.9)
Flaking	305 (79.4)
Scales	269 (70.1)
Pain	209 (54.4)
Bleeding	199 (51.8)
Burning/stinging	184 (47.9)
Nail separation, crumbling, or discoloration	60 (15.6)

Abbreviations: PsA, psoriatic arthritis; PsO, psoriasis; UPLIFT, Understanding Psoriatic Disease Leveraging Insights for Treatment.

^a^ Patient‐reported health‐care‐provider diagnosed osteoarthritis or rheumatoid arthritis in UPLIFT.

^b^ Crohn's disease and ulcerative colitis.

^c^ Among patients with PsO, and presence of skin symptoms (*n* = 384); note that a patient may have PsO in >1 location.

^d^ Among patients with PsO, and presence of skin symptoms (*n* = 384); note that a patient may have >1 PsO symptom.

### 3.2 Patient demographics and dermatologist practice characteristics

The mean age of Japanese patients was 46.6 years, and 58% of patients were men (Table 1). The most commonly reported comorbid conditions were hypertension (36%), depression (28%), arthritis (25%), and diabetes (23%). Cancer occurred in approximately 21% of patients, as did liver disease.

Among the 51 Japanese dermatologists who completed the survey, 63% practiced in a hospital‐based setting and 37% in a community‐ or office‐based setting. Japanese dermatologists reported that they spent most of their time (90%) treating patients. They treated a mean of 633 patients per month in their practices (mean of 41 with PsO; mean of 15 with PsO and PsA); 88% of their patients with PsO had plaque PsO.

### 3.3 Patient‐rated disease characteristics

**FIGURE 1:**
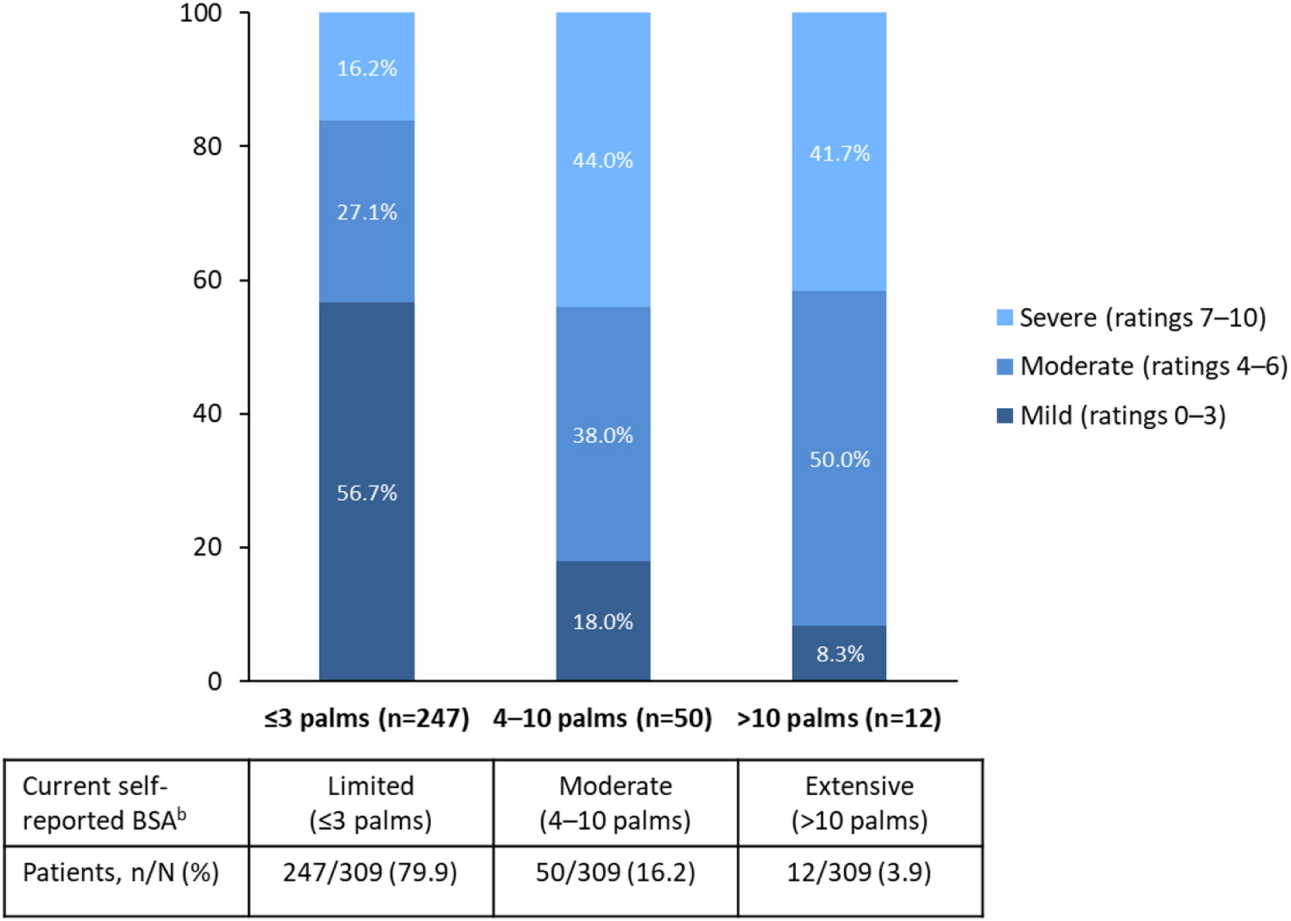
Severity of current PsO symptoms by self‐reported BSA category (assessed by number of palms)^a^. ^a^In response to the question: On a scale of 1–10, where 1 = “very mild” and 10 = “very severe”, please tell us… a. How severe is your psoriasis currently? ^b^In response to the question: Based on the amount of PsO that could be covered by the palm of your hand (including fingers), how many palms of PsO would you say you currently have? BSA, body surface area; PsO, psoriasis

Body surface area data were available for 309 Japanese patients currently experiencing PsO symptoms. The majority of these patients (80%) reported BSA involvement of ≤3 palms (Figure 1). Among patients with BSA ≤3 palms, 43% rated their current PsO symptoms as moderate or severe. In patients with BSA 4–10 palms, symptoms were considered to be moderate by 38% of patients and severe by 44%.

**FIGURE 2:**
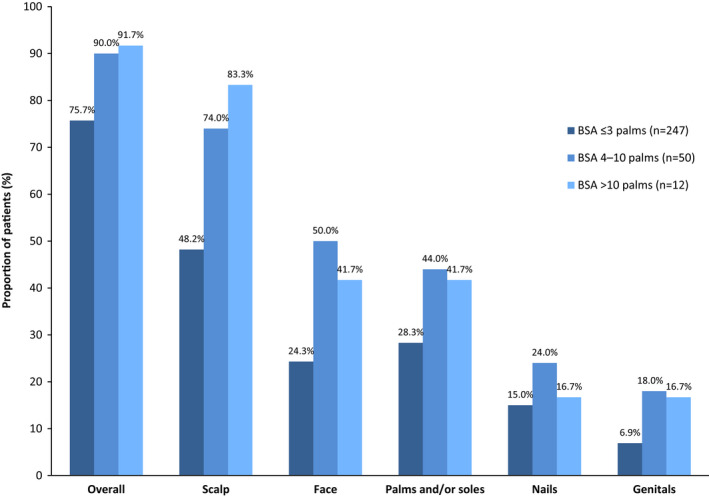
Special area involvement according to self‐reported BSA categories.BSA, body surface area

Patients commonly reported PsO involvement in special areas. Of the 384 patients with PsO (with or without PsA), 48% reported scalp involvement and 26% reported PsO on the face (Table 1). Palms, soles, nails, and genitals were affected in up to 20% of patients. PsO occurred in at least one special area in 76% of patients with BSA ≤3 palms and in ≥90% of those with BSA >3 palms (Figure 2). Scalp involvement was the most common location of special interest in all BSA subgroups (48%–83%) and genital involvement was the least common (2%–18%).

The most common PsO symptom reported by patients with PsO (with or without PsA) was itching (91%), followed by redness (86%), flaking (79%), and scales (70%) (Table 1). Pain and bleeding occurred in 54% and 51% of patients, respectively.

Among the 293 patients with PsO alone, 122 (42%) reported experiencing joint discomfort, and 87 (71%) of these patients had ≤4 involved joints, consistent with oligoarthritis. Regarding musculoskeletal symptoms suggestive of PsA in patients with PsO alone, 23% reported morning joint stiffness, 30% reported nail involvement, 19% reported pain and/or swelling of the feet, and 10% reported evidence of “sausage” digits; 46% and 7% of patients responded “none of the above” and “not sure”, respectively. Overall, the PEST score was ≥3 in 32% of patients who experienced joint discomfort, indicating that referral to a rheumatologist should be considered.^24^ The PEST score was ≥3 in 24% (*n* = 87) of patients with ≤4 affected joints and 51% (*n* = 35) of patients with >4 affected joints.

Patients with PsA and/or PsO (*n* = 98) had a mean HAQ‐8 score of 0.8.

### 3.4 Patient‐reported current treatment

**FIGURE 3:**
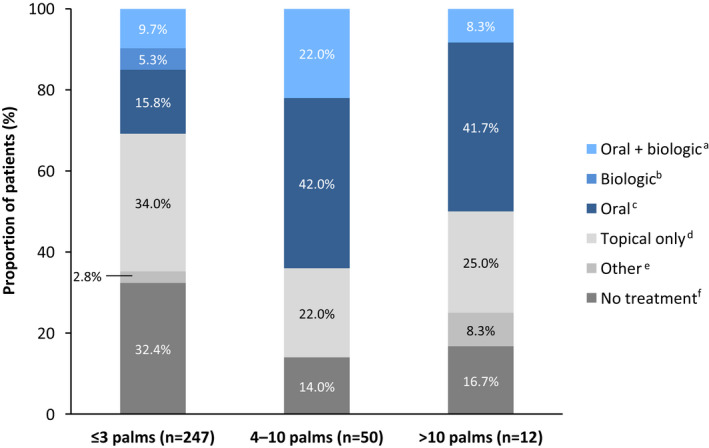
Current PsO treatment by level of BSA involvement (assessed by number of palms) in patients with PsO (with or without PsA) currently experiencing PsO symptoms. ^a^Oral prescription + biologic or topical + oral prescription + biologic. ^b^Biologic only or biologic + topical prescription. ^c^Oral prescription only or oral + topical prescription. ^d^Topical prescription only. ^e^Other only, phototherapy only, phototherapy + other (i.e., anything other than prescription oral/biologic/topical therapy or phototherapy). ^f^No treatment other than oral or topical OTC therapy. BSA, body surface area; OTC over‐the‐counter; PsA, psoriatic arthritis; PsO, psoriasis

As shown in Figure 3, most patients with PsO and/or PsA with BSA involvement of ≤3 palms (*n* = 247) reported receiving topical therapy alone (34%) or no treatment (32%); only 31% of patients reported receiving systemic therapy, including oral, biologic, or oral + biologic therapy. Among patients with BSA ≥4, 50% (>10 palms) to 64% (4–10 palms) of patients were receiving systemic therapy (i.e., oral or biologic therapy); however, substantial proportions of patients were not receiving any treatment or only topical therapy.

### 3.5 Patient‐reported quality of life

**FIGURE 4:**
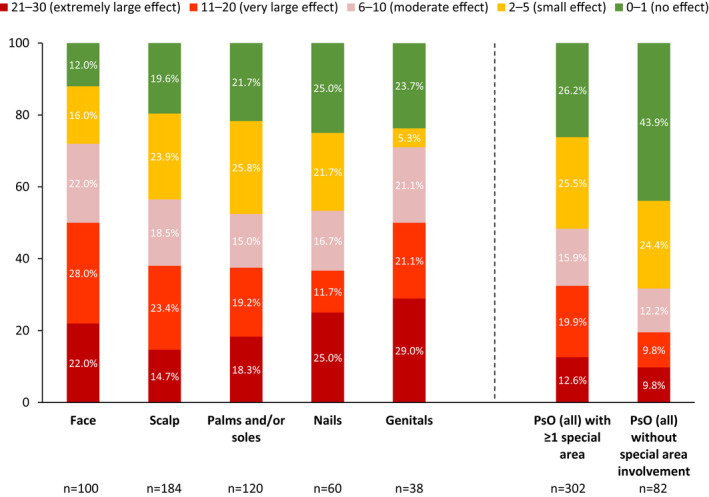
DLQI total score categories by special area involvement. Patients could have involvement in >1 special area. Brackets show patients reporting at least a moderate effect. DLQI, Dermatology Life Quality Index; PsO, psoriasis

Overall, in patients with PsO with or without PsA (*n* = 384), 45% of patients experienced at least a moderate impact on QoL (DLQI score >5). Patients with PsO involvement in at least one special area tended to have higher DLQI scores than patients with no special area involvement (Figure 4). DLQI scores >5 occurred in 71%–72% of patients with face or genital involvement and 53%–57% of patients with PsO in other special areas.

**FIGURE 5:**
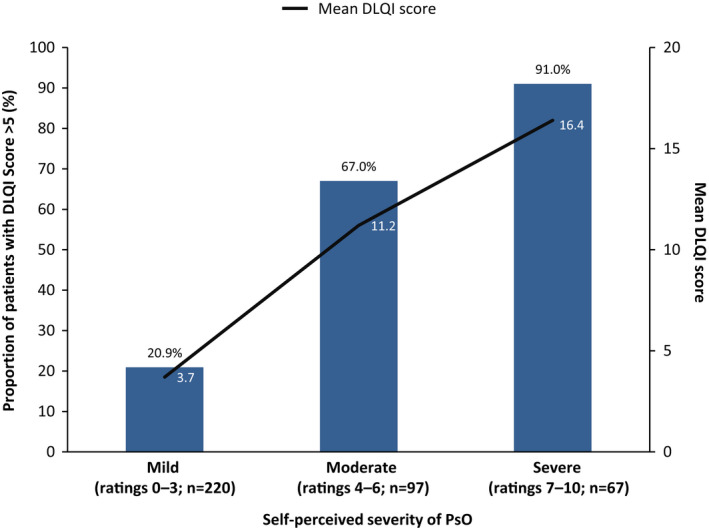
DLQI scores >5 by self‐perceived severity of PsO (mild‐to‐severe). DLQI, Dermatology Life Quality Index; PsO, psoriasis

As shown in Figure 5, the impact of PsO on QoL was highest in patients with self‐perceived severe disease based on a visual analog scale, but 21% of patients with self‐perceived mild disease still had a DLQI score >5. Mean DLQI score ranged from 3.7 in patients with self‐perceived mild disease to 16.4 in those with self‐perceived severe disease.

### 3.6 Patient and dermatologist perceptions of disease burden and office visits

**FIGURE 6:**
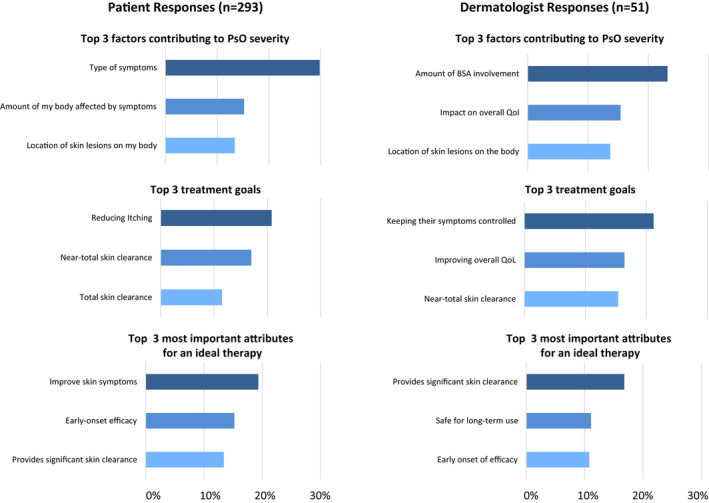
Patient and dermatologist perceptions of determinants of PsO severity and treatment. Percentages were calculated by dividing the sum of scores by the maximum possible score. Separate responses were provided regarding patients in different severity categories (mild, moderate, or severe in the dermatologists' estimation). The aggregate of the three categories is shown. BSA, body surface area; PsO, psoriasis

Patients with PsO ranked the type of symptoms as the most important factor contributing to PsO disease severity, followed by the amount of the body affected by symptoms (Figure 6). In contrast, dermatologists considered the amount of BSA involvement to be the most important factor contributing to disease severity in their patients with PsO, followed by impact on QoL. Dermatologists and patients (13%–14%) both considered the location of skin lesions to be the third most important factor contributing to disease severity.

### 3.7 Patient and dermatologist perceptions of treatment goals and attributes

While the most important goal of treatment was reduced itching for patients with PsO, dermatologists considered keeping their patients' symptoms controlled as their top priority, without focusing on any particular symptom (Figure 6). Skin clearance was considered important by both patients and dermatologists, whereas only dermatologists considered improving overall QoL as one of their top three treatment goals.

Patients considered improving skin symptoms to be the most important attribute for an ideal therapy, whereas dermatologists rated significant skin clearance as the top treatment attribute (Figure 6). Patients also considered early‐onset of efficacy and provision of significant skin clearance to be important treatment attributes. In addition to early‐onset of efficacy, dermatologists considered long‐term safety to be a top attribute.

### 3.8 Patient and dermatologist perceptions of office visits

**FIGURE 7:**
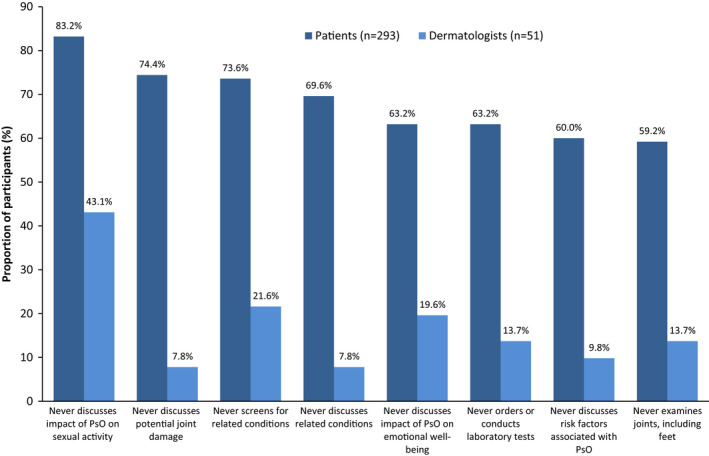
Patient and dermatologist perceptions of office visit discussions. PsO, psoriasis

When recalling office visits, considerably more patients than dermatologists reported never discussing potential joint damage, related conditions, and the impact of PsO on emotional well‐being (Figure 7).

## 4 DISCUSSION

The current report reflects the findings from Japanese participants in the UPLIFT survey. To our knowledge, this is the first survey describing different aspects of PsO – signs/symptoms, QoL, and treatment patterns – in Japanese patients with PsO, especially with limited skin involvement. The results confirm that Japanese patients with PsO can experience a substantial disease burden, irrespective of the level of skin involvement, and that BSA often underestimates PsO severity, resulting in undertreatment. In line with the overall findings of the UPLIFT survey,^11^ the Japanese subgroup analysis identified a need for better management of PsO, and several areas in which patients' and dermatologists' perceptions are not aligned.

The prevalence of PsO varies globally, but is generally lower in Asia than in other parts of the world.^1^, ^15^ The population prevalence of self‐reported PsO and/or PsA in the Japanese UPLIFT subgroup (2%) was higher than the estimated prevalence of diagnosed PsO and/or PsA previously reported in a nationwide study using the Japanese national claims database (0.3%),^25^ and lower than that reported in the overall UPLIFT survey (4%).^11^ Although the reasons underlying this difference in prevalence are unknown, one possible explanation could be differences in the methods used for study conduct. While the Japanese national claims database included only patients with hospital medical records, the UPLIFT survey was conducted online and recruited patients who self‐reported a health‐care provider diagnosis of PsO and/or PsA.

Most Japanese patients in the UPLIFT survey reported having PsO alone (75%) or with PsA (23%), with only 2% of patients having PsA alone. This is consistent with the recognized pattern of PsO generally preceding or occurring concurrently with PsA.^1^, ^26^ As observed in the Western Japan Psoriasis Registry (WJPR) study, in which 27% of patients had PsA,^17^ Japanese patients from the UPLIFT survey had a higher prevalence of PsA than that observed in the Fukuoka University Psoriasis Registry study (17%).^27^ Furthermore, 42% of patients with a diagnosis of PsO alone experienced joint discomfort affecting ≤4 joints, which was therefore consistent with oligoarticular PsA.^28^ The PEST score, which can be used to detect PsA in Japanese patients,^29^ indicated that 32% of Japanese patients in UPLIFT with PsO alone and joint discomfort should be managed with a multidisciplinary approach in collaboration with rheumatologists. To identify PsA early, it is important that patients with PsO are frequently questioned about arthritic symptoms such as joint pain, morning stiffness, and dactylitis.^28^ When Japanese patients in UPLIFT responded to questions about such symptoms, 19%–42% of patients with PsO alone indicated that they had one of these symptoms suggestive of PsA.

In addition to PsA, PsO is associated with systemic inflammation and other comorbidities that further increase the burden of disease, including cardiometabolic disease.^1^, ^2^ In this Japanese subgroup analysis of UPLIFT, the prevalence of hypertension, diabetes, and heart disease were high, affecting 36%, 23%, and 18% of patients, respectively. Hypertension was also the most common comorbidity in the WJPR study and the Japanese Society for Psoriasis Research (JSPR) survey (≥30% of patients), followed by diabetes (≥15% of patients).^15^, ^17^ Cancer and liver disease were relatively common in Japanese patients in UPLIFT (each occurring in ~21% of patients compared with 8% and 11% of patients in the WJPR study, respectively),^17^ and may have influenced PsO treatment choice in favor of topical treatment.^26^

Patients with PsO are also known to be at increased risk of depression,^1^, ^2^ which was reported by 28% of Japanese patients in UPLIFT. Depression/anxiety disorder was less common in the WJPR analysis, occurring in only 7% of patients,^17^ and was not reported in the JSPR survey.^15^ Compared with these physician‐led surveys, it is possible that patients recruited online to the UPLIFT survey were more aware of their mental health status, and therefore were more likely to respond in the affirmative for comorbid depression. It is also possible that the coronavirus disease 2019 (COVID‐19) pandemic, which was ongoing during the period in which the survey was conducted, may have contributed to the patients' emotional burden.

According to palm‐based BSA involvement, which is one of the most commonly used measures to assess the severity of PsO (where one palm width represents ~1% of BSA),^6^ the majority (80%) of the 309 Japanese patients with self‐reported BSA data participating in UPLIFT had mild disease (≤3% BSA involvement), 16% had moderate disease (4%–10% BSA), and 4% had severe disease (>10% BSA). In a recently published analysis of data from the WJPR (September 2019 to December 2020; *n* = 1349), a similarly high proportion of patients (76%) had BSA involvement of ≤3%.^17^ It was thought that this may reflect the superior efficacy of the latest systemic drugs.^17^ The most frequently used oral drug in the WJPR study was apremilast (18.0%), followed by methotrexate (8%), etretinate (4%), and cyclosporin (4%), and the most frequently used biologics were interleukin (IL)‐17 inhibitors (32%), followed by IL‐23 inhibitors (27%) and tumor necrosis factor inhibitors (11%).^17^

Irrespective of the level of skin involvement, PsO occurred in special areas of the body in the majority of Japanese patients in the UPLIFT survey, including 76% of those with BSA involvement of ≤3 palms. Scalp and face PsO were the most common special areas, affected in 48% and 26% of patients, respectively, and genital involvement was the least common, reported in 10% of patients. In a previous epidemiologic survey of >15 000 Japanese patients with PsO conducted by the JSPR from 2013 to 2018, the proportions of patients with a BSA involvement of <5%, 5%–10%, and >10% were 37%, 27%, and 36%, respectively, and similar to the Japanese patients in UPLIFT, PsO affecting the scalp, face, or genitals was reported in 50%, 31%, and 14% of patients, respectively.^15^

In the final 2 years of the JSPR study, use of topical therapies declined and the use of newly approved oral and biologic agents increased, with apremilast becoming the most commonly administered oral medication from 2017 onwards.^15^ In 2018, the anti‐IL‐23 p19 antibody guselkumab overtook IL‐17 inhibitors as the most commonly used biologic.^15^ Despite the expanding number of therapeutic options for managing PsO,^9^, ^16^ and the high disease burden in a population with limited skin involvement, the UPLIFT survey results suggest that many Japanese patients are not treated or are undertreated. Although a substantial proportion of patients with mild PsO (according to BSA involvement) rated their current symptoms as moderate or severe (43%), approximately 66% of these patients were receiving no therapy or topical therapy alone. Additionally, despite >80% of patients with more extensive skin involvement having self‐perceived moderate or severe symptoms, 36–42% of these patients were receiving topical therapy alone or no treatment.

Although the BSA involvement is an indicator of the severity of PsO, it does not capture other variables such as PsO location, symptom intensity, comorbidities, or QoL,^6^, ^13^ and patient‐ and physician‐reported severity and treatment satisfaction are often mismatched, which was also highlighted in both the MAPP and UPLIFT surveys.^5^, ^11^, ^22^, ^23^, ^30^, ^31^ Difference in perceptions may explain why some PsO patients, who may benefit from suitable systemic therapy, still remain only on topical therapy.^11^ To avoid underestimating disease severity and consequently undertreating, comprehensive care of PsO patients with mild‐to‐moderate skin involvement should take into account the location of lesions and impact of PsO on the patient's QoL, as well as assessing BSA involvement.^8^, ^9^, ^13^, ^26^, ^32^ In support of this, the impact of PsO on patient QoL in the overall UPLIFT study population,^11^ and in the Japanese subgroup was higher in patients with special area involvement. Particularly high proportions of Japanese patients with facial or genital PsO experienced at least a moderate effect of PsO on their QoL (71%–72%), confirming that PsO in these areas is disproportionately distressing. These findings are consistent with a prior study showing a relationship between PsO in visible and sensitive special areas and QoL impairment.^33^

Previous research in Japanese patients suggests that persistence of genital PsO plays an important role in inadequate treatment satisfaction, possibly because physicians are not aware of symptoms in this area, and patients tend to have higher expectations of treatment than physicians (i.e., complete clearance).^22^, ^23^ Indeed, recall of past office visit discussions differed between Japanese patients and dermatologists in the UPLIFT survey, highlighting that better alignment of patient and physician perceptions of PsO treatment and goals of therapy is needed. In the UPLIFT survey, the location of skin lesions was one of the three most important factors contributing to disease severity reported by Japanese patients with PsO and dermatologists alike (Figure 6). However, patients and dermatologists tended to assess disease severity differently, with patients appraising the type of symptoms as the most important factor contributing to disease severity, while dermatologists placed the most importance on the amount of BSA involvement. Although patients chose total skin clearance as one of the top treatment goals, our results suggest that patients would be satisfied with near‐total skin clearance. Reducing the commonly reported symptom of itching was the most important treatment goal for patients, and improving skin symptoms was the most important treatment attribute. Keeping patients' symptoms controlled was the top treatment goal of dermatologists, but the ability of a treatment to provide significant skin clearance was deemed to be its most important attribute. Interestingly, the MAPP survey also highlighted a discrepancy between how patients and dermatologists perceived PsO disease severity.^5^, ^31^ These observations suggest a need for Japanese dermatologists to recognize the importance of assessing and managing other bothersome symptoms of PsO, such as pruritis, as well as focusing on skin clearance while managing patients' expectations.

This analysis had some limitations that are inherent to surveys, such as recall bias from patients with a self‐reported health‐care provider diagnosis of PsO or PsA. Participants were selected from an online panel of adults that may not be fully representative of the general population; for example, patients who are familiar with the internet tend to respond to such online surveys. The sample of dermatologists was intended to be representative of physicians who regularly treat patients with PsO but did not necessarily include specialists who exclusively treat PsO, and therefore the included dermatologists may not have been sufficiently specialized in the treatment and management of PsO. Furthermore, findings from this Japanese subgroup analysis are limited by the relatively small number of patients, particularly those with extensive PsO (BSA involvement of >10 palms; *n* = 12), whose responses may not be representative of the overall Japanese population of patients with extensive PsO. In addition, as part of this survey was conducted during the COVID‐19 pandemic, the emotional burden associated with COVID‐19 may have had an impact on psychological and physiological aspects of living with PsO and could have affected assessments of patient‐reported outcomes. Pandemic restrictions may also have limited in‐office visits and necessitated reliance on virtual visits, which could have affected patient and physician responses. Meanwhile, a strength of UPLIFT is that it was not limited by current medical care and participant identification was not based on membership of any patient organization or health‐care provider care, so participants were likely to be more broadly representative of the cross‐section of individuals living with PsO in Japan.

In conclusion, findings from the 2020 UPLIFT survey highlight a persistent unmet treatment need in Japanese patients with PsO who perceive their current symptoms to be moderate or severe regardless of the level of skin involvement, particularly in patients with PsO in special areas. Nearly two‐thirds of Japanese patients in UPLIFT were receiving only topical therapy or no treatment, despite a high proportion of patients perceiving their symptoms to be moderate or severe and many patients reporting substantial disease burden, bothersome symptoms, joint involvement, and PsO involvement in special areas. This suggests a need for increased use of appropriate systemic therapy in Japanese patients with PsO. Overall, these findings highlight an opportunity to enhance patient–dermatologist relationships to improve outcomes and address the unmet needs of patients with PsO across the disease severity continuum.

## CONFLICT OF INTEREST

H.T. received consulting fees and/or honoraria from the following companies: AbbVie, Amgen, Eli Lilly, Janssen, Kyowa Kirin, Novartis, Sun Pharma, and UCB Japan. K.M. received consulting fees and/or honoraria from the following companies: AbbVie, Amgen Astellas BioPharma, Asahi‐Kasei Pharma, Astellas, Ayumid Pharma, BMS, Chugai, Daiichi Sankyo, Eisai, Gilead, Janssen, Kyowa Kirin, Lilly, Novartis, Ono Pharma, Pfizer, Tanabe‐Mitsubishi, Teijin Pharma, and UCB. M.T., H.N., and S.C. received salaries from Amgen K.K. as its employees.
